# Analysis of Patient Safety Incident reporting system as an indicator of quality nursing in critical care units in KwaZulu-Natal, South Africa

**DOI:** 10.4102/hsag.v25i0.1263

**Published:** 2020-03-31

**Authors:** Thusile M. Gqaleni, Busisiwe R. Bhengu

**Affiliations:** 1 School of Nursing and Public Health, University of KwaZulu-Natal, Durban, South Africa

**Keywords:** adverse events, quality patient care, harm, patient safety, critical care unit

## Abstract

**Background:**

Patient Safety Incidents occur frequently in critical care units, contribute to patient harm, compromise quality of patient care and increase healthcare costs. It is essential that Patient Safety Incidents in critical care units are continually measured to plan for quality improvement interventions.

**Aim:**

To analyse Patient Safety Incident reporting system, including the evidence of types, frequencies, and patient outcomes of reported incidents in critical care units.

**Setting:**

The study was conducted in the critical care units of ten hospitals of eThekwini district, in KwaZulu-Natal, South Africa.

**Methods:**

A quantitative approach using a descriptive cross sectional survey was adopted to collect data from the registered nurses working in critical care units of randomly selected hospitals. Self-administered questionnaires were distributed to 270 registered nurses of which 224 (83%) returned completed questionnaires. A descriptive statistical analysis was initially conducted, then the Pearson Chi-square test was performed between the participating hospitals.

**Findings:**

One thousand and seventeen (*n* = 1017) incidents in ten hospitals were self-reported. Of these incidents, 18% (*n* = 70) were insignificant, 35% (*n* = 90) minor, 25% (*n* = 75) moderate, 12% (*n* = 32) major and 10% (*n* = 26) catastrophic. Patient Safety Incidents were classified into six categories: (a) Hospital-related incidents (42% [*n* = 416]); (b) Patient care-related incidents (30% [*n* = 310]); (c) (Death 12% [*n* = 124]); (d) Medication-related incidents, (7% [*n* = 75]); (e) Blood product-related incidents (5% [*n* = 51]) and (f) Procedure-related incidents (4% [*n* = 41]).

**Conclusion:**

This study’s findings indicating 1017 Patient Safety Incidents of predominantly serious nature, (47% considering moderate, major and catastrophic) are a cause for concern.

## Introduction and Background

The high occurrence of Patient Safety Incidents (PSIs) leading to preventable deaths remains a global concern (Bashir et al. 2019; Guillod 2013). In the United States, PSIs were recently estimated to cause up to 98 000 preventable deaths each year (James 2013). This high rate of PSIs has attracted significant attention from the public, medical providers and health care payers (Bauman & Hyzy 2014; Gonçalves et al. 2012; James 2013; Wassenaar, Schouten & Schoonhoven 2014). According to James (2013), and Wassenaar et al. (2014), there is a rising global concern that approximately 400 000 patients per year suffer from preventable harms that contribute to death. Medical errors have been highlighted as the major contributing factors to PSIs and to patients’ morbidity and mortality in the United States and United Kingdom (Sommella et al. 2014). About 30% to 70% of patients admitted to hospitals experience PSIs that could have been prevented (Montenegro et al. 2016). In addition, population-based studies from a number of countries have consistently demonstrated unacceptably high rates of medical injury and preventable deaths (Gonçalves et al. 2012; Gong et al. 2015; West & Eng 2014). Patient Safety Incidents have an impact on quality patient outcome, which may result in human cost, for example, patients’ complaints, suffering, permanent incapacity or even death (Ramya 2017; Roque, Tonini & Melo 2016).

South Africa is among the developing countries that have a higher percentage of PSIs because of increased disease burden, aggravated by the human immunodeficiency virus (HIV) infection and acquired immune deficiency syndrome (AIDS) pandemic, which have a profound impact on critical care units (CCUs), resulting in complicated illnesses. Naidoo, Singh and Lalloo (2013) confirm that these patients develop opportunistic infections, which require specialised care in CCUs, with resources allocated that rarely match the demand. Most of the patients are admitted with severe illnesses and comorbid diseases, making them susceptible to PSIs (Naessens et al. 2012). According to Naessens et al. (2012), sicker patients often require more frequent, intensive and complex treatment, exposing them to increased occurrence of PSIs, thus increasing morbidity and mortality rates. Moreover, Welters et al. (2011) observed that in the United Kingdom, the contributing factors to PSIs range from shortage of critical care facilities (shortage of CCUs beds), shortage of skilled, knowledgeable staff, increasing workload and faulty equipment. In South Africa, these resources are much more limited than in developed countries, yet the impact of error on patient outcome is just as important (Bashir et al. 2019).

## Statement of the research problem

There are much more PSIs among critically ill patients that contribute to harm, resulting in exorbitant health care costs compared to the patients in general care (Bauman & Hyzy 2014; Guillod 2013), yet PSIs remain underreported (Gong et al. 2015; Hooper & Tibballs 2014; Schultz et al. 2014). In South Africa, a study conducted by Mjadu and Jarvis (2018) on patients’ safety in adult CCUs revealed that irrespective of high percentages of occurrence of PSIs, these PSIs remain underreported, and in the case of reported PSIs, no measures to prevent recurrence are taken. One of the barriers, a contributing factor to this underreporting, is increase in malpractice claims, and fear of punitive action, which discourages voluntary reporting (Pepper & Slabbert 2011). Thus, this underreporting does not encourage the PSIs reporting culture in CCUs, prevents learning from the feedback and hence increases the chances of their recurrence, which stifles improvement in quality patient care.

According to Pepper and Slabbert (2011), measuring the frequencies and severities of PSIs in CCUs is important for quality improvement interventions. Documentation is emphasised as important to prevent PSIs, not only because of the impact thereof on patients but also because the documentation of information may provide an insight into the quality of healthcare and an opportunity for improvement (Hooper & Tibballs 2014). On the other hand, Heavner and Siner (2015) argue that PSIs occur from the inappropriate delivery of health care or from an error; hence, they define medical errors as a result of failure of a planned action to be completed as intended (error of execution) or from the use of an incorrect plan to achieve a goal (error of planning).

According to the World Health Organisation (WHO 2005), the most important knowledge in the field of patient safety is how to prevent harm to patients during treatment and care; hence, the currency of patient safety can only be measured in terms of harm prevented and lives saved. This was further reiterated on the revised WHO (2017) guidelines for Patient Safety Incident Reporting and Learning System (WHO 2017). In addition, Tysall and Duffy (2013) stated that the need to use more rigorous processes to ensure that health care recommendations are informed by the best available research evidence. Moreover, Archer et al. (2017) confirm that to ensure patient safety, the development and implementation of Patient Safety Incident Reporting System within health care continues to be a fundamental strategy to reduce preventable patient harm and improvement in the quality of patient care. Therefore, in this study, the national PSI reporting system related to patient safety, which is in line with recommendations of the WHO, was analysed.

The South African National Department of Health (NDoH) has shown more commitment to improving the quality of health care. Aligning itself with the WHO recommendations, South Africa formulated *National Guidelines for Patient Safety Incident Reporting and Learning System* to guide the health care system in dealing with patient safety incident reporting (NDoH 2017). Although the KwaZulu-Natal Department of Health (KZN DoH) has formulated guidelines on the reporting of types, frequencies and severities of PSIs, it is not clear whether there is evidence of the reduction in PSIs. Hence, the aim of the study was to analyse the PSIs reporting system, including the types, frequencies and patient outcomes of reported PSIs in CCUs.

## Definition of key concepts

*Patient Safety Incidents* are incidents related to medical mismanagement that result in harming patients in contrast to disease complications or underlying disease (WHO 2017). These PSIs occur in CCUs and result in lengthy stay in hospitals. In this study, various types of PSIs that occur in CCUs are found and classified.

*Quality patient care* refers to the extent to which an organisation meets its client’s needs and expectations. It is a complex multifaceted concept measured against predetermined standards (Whittaker et al. 2011). PSIs occur as a result of medical errors, which may be errors of commission or omission and usually reflect deficiencies in the system of care. In this study, quality patient care refers to safe patient care and treatment, in relation to occurrence of PSIs, their outcomes and reporting.

*Harm* refers to impairment of structure, or body function or any deleterious effect, resulting from or contributed to by medical care that requires additional monitoring, treatment or hospitalisation, or that results in death (WHO 2017). In this study, harm meant unintended harm sustained by the patient during medical care.

*Patient safety* means freedom from accidental injuries or harm. Initiatives are designed to prevent adverse outcomes from medical errors (Ramya 2017). In this study, patient safety refers to a safe environment for patients in CCUs in relation to occurrence of PSIs, their outcomes and reporting.

*Critical care unit* is a specialised hospital environment where critically ill and injured patients are nursed and treated (Heavner & Siner 2015). It is staffed with specialised nurses and equipped with complex technology for monitoring care and treatment of patients with life-threatening conditions, for example, neonatal, cardiac and cardiothoracic CCUs. Critical care units are named differently by different hospitals such as surgical, medical, general, surgical trauma, neuro or burns, but in this study, these are grouped into multidisciplinary CCUs.

## Research methodology

### Research design

The research design was a descriptive, non-experimental, cross-sectional survey. This was a quantitative approach, within a bigger mixed methods study, which used a concurrently embedded strategy. A self-administered questionnaire was used to collect data on types, frequency and patient outcomes of reported PSIs in CCUs and how they are managed.

### Study area

The study was conducted in eThekwini district, KwaZulu-Natal (KZN), South Africa, the largest district in the province, with most hospitals having CCUs and diverse patient profiles, referred from rural as well as semi-urban areas and from other provinces. A fishbowl technique was used (Brink, Van Der Walt & Van Rensburg 2012), involving listing of eThekwini hospitals with CCUs and assigning them numbers. They were then written on small papers and put in a bowl from which they were randomly picked until the determined 10 hospitals were selected. The sample frame consisted of 28 hospitals; hence data were collected in 10 out of 28 hospitals with various types of CCUs. A multistage sampling was applied using a Raosoft sample size calculator, and a sample size of 10 was found to be sufficient to yield valid results. These included four major hospitals from the public sector and six from the private sector. The number of CCUs in this study totalled 32, because some hospitals had more than one CCU.

### Population

The target population comprised all 700 registered nurses (RNs) working in the selected CCUs, of which 38.5% were selected as sample and categorised as follows: 179 direct care or bedside nurses, who had CCU speciality; 47 non-specialists; and 44 operational managers, totalling 270.

### Sample and sampling

The sample size consisted of 38, 5% of 700 RNs, therefore, of which 270 were extracted. Convenient sampling was used, based on accessibility of the participants who consented and met inclusion and exclusion criteria. For power analysis, Raosoft sample size calculator was used and a sample size of 249 was found to be sufficient to yield valid results. The inclusion criteria were the RNs who had worked in CCUs with critical care speciality, including those who did not have the speciality but had CCU experience, and operational managers. The RNs who met the inclusion criteria and consented had more than one year of experience in CCUs. Registered nurses, who worked in CCUs but had less than one year of CCU experience, or did not work in CCUs and those who were not willing to participate in the study were excluded.

### Research instrument

The researcher modified KZN DoH guidelines for PSIs monitoring and reporting into a questionnaire. These guidelines are in the public domain, prepared by the Health Outcomes Research Unit and the Evaluation Unit of KwaZulu-Natal Department of Health (Mahomed, Moodley & Jinabhai, n.d.). The questionnaire had closed-ended questions divided into four sections. Section A addressed respondents’ demographics, namely types of CCUs, respondents’ level of experience and job level; section B addressed the existence and utilisation of the reporting system; and section C addressed types and frequency of PSIs. Section D addressed the six categories of PSIs, namely patient-related, procedure-related, death medication-related blood product-related and procedure-related categories. Section E addressed the patient outcomes indicating the severity of these PSIs.

Validity and reliability were ensured through pretesting the instrumentation on a sample of 10 RNs, who worked in CCUs and studying specialisation in critical care nursing at the university. The sample comprised both RNs and unit managers who met the inclusion criteria of having worked in CCUs for more than a year. Pretesting of the questionnaire was to ensure clarity and meaning of presented concepts and simplicity of statements. Only section B was added to the guidelines and the respondents suggested changes in three items, in terms of simplicity of language and sequence of items. These suggestions were then incorporated into the final instrument. These respondents did not form part of the final sample, and data collected during this process were not included in the main study. Materials and procedures were not modified as a result of pilot data. A content validity was also performed, whereby the items of the research instrument were compared with the objectives of the study to ensure that the tool was measuring what it purported to measure. Using Statistical Package for the Social Sciences (SPSS), the Cronbach’s alpha coefficient was also calculated on all the items of the instrument, and a value of 0.9 (*p* = 0.9) indicated a good internal consistency, that is, the items measured the same construct.

### Data collection process

After securing ethics clearance from the Human Science Research Ethics Committee of the University of KwaZulu-Natal (reference HSS/1554/015D) and permission from the participating hospitals, the researcher held scheduled meetings with the management of these hospitals. The research purpose, process, participants’ rights and potential risks, including their mitigation, were explained, and signed informed consents were secured. The self-administered questionnaire was distributed to 270 RNs who worked in CCUs. Each participant was asked to complete the questionnaire and deposit it into a sealed, labelled box that was left in the unit manager’s office. The completed questionnaires were collected from each participating institutions every 2 weeks over a period of 3 months, from December 2015 to February 2016.

### Data analysis

The SPSS version 21 was used to analyse the data. Data analysis was initiated with a check of data for outliers, missing data and normality through skewness and kurtosis values that could affect relations between variables. The descriptive statistical analysis of the data (means, standard deviations, ranges, frequencies and percentages) was initially conducted prior to conducting the Pearson’s chi-square test of association between any categorical variables, including differences between participating hospitals, both the government and private hospitals. Data were also analysed to explain the correlations of RNs’ experience and job level, types, frequency and prevalence of PSIs, as well as patient outcomes, indicating the severity of these PSIs.

### Ethical consideration

Ethical approval to conduct the study was obtained from the Humanities and Social Science Research Ethics Committee at the University of KwaZulu-Natal (clearance number: HSS-1554-015D).

## Results

### Demographic characteristics of participants

The number of questionnaires returned was 224, making a return rate of 83%. The distribution of the returned questionnaires is as follows: 76% (*n* = 171) from the RNs who had an additional qualification in critical care nursing, 11% (*n* = 25) was from the RNs who did not have any speciality and 13% (*n* = 28) from operational managers. All the participants had the CCU experience ranging from 4 to 39 years, with a mean of 6.7 years, indicating that most of the respondents had an in-depth experience of CCUs.

The distribution of participants according to CCUs they were working in is presented in Table 1, which demonstrates that the majority (130 [58%]) of RNs were from multidisciplinary CCUs, which included general, surgical CCUs, neuro or burns CCUs, surgical trauma and medical CCUs, followed by neonatal CCUs (46 [20.5%]) and cardiac CCUs (37 [16.5%]); the latter included coronary care and cardiothoracic CCUs. The least number of participants (11 [4.9%]) were from paediatric CCUs.

**TABLE 1:** Number of registered nurses’ responses from different types of critical care units in the selected hospitals of eThekwini, KwaZulu-Natal.

CCU type	*n*	%
Multidisciplinary CCU	130	58.0
Neonatal CCU	46	20.5
Cardiac	37	16.5
Paediatric CCU	11	4.9
**Total**	**224**	**100**

CCU, critical care unit.

### Existence and utilisation of reporting system

The majority of participants (*n* = 218 [97%]) indicated that the reporting system existed in their units, 39% used web-based system, while 37% used written documentation. The majority of participants (*n* = 189 [84%]) indicated that they had utilised the reporting system, while 16% (*n* = 35) reported not having used it. The reasons for non-utilisation ranged from non-reaction to report (*n* = 10 [4%]), fearfulness (*n* = 17 [9%]) and busy schedules (*n* = 8 [3%]). The majority (70%) of participants who reported non-utilisation were from public hospitals. Moreover, the majority of participants (*n* = 159 [71%]) indicated that PSIs were not managed, and 18% (*n* = 41) did not know whether PSIs were managed or not.

### Types and frequencies of Patient Safety Incidents

**FIGURE 1:**
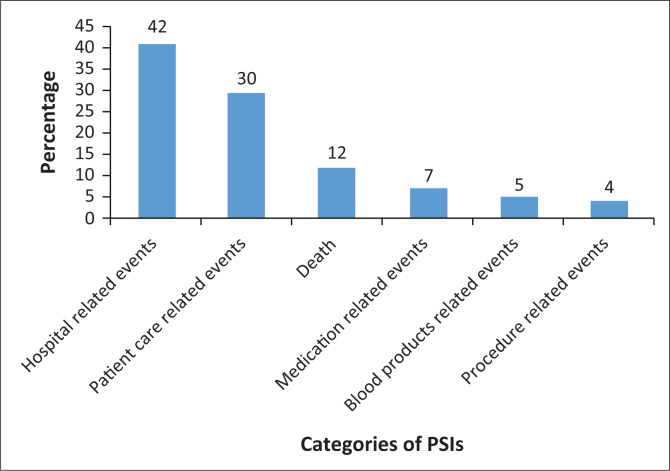
Distribution of major Patient Safety Incidents within six categories.

In this study, PSIs were classified into six major categories, as illustrated in Figure 1: (1) hospital-related incidents (*n* = 416 [42%]); (2) patient care-related incidents (*n* = 310 [30%]); (3) death (*n* = 124 [12%]); (4) medication-related incidents (*n* = 75 [7%]); (5) blood product-related incidents (*n* = 51 [5%]) and (6) procedure-related incidents (*n* = 41 [4%]).

The study also included all the PSIs for a period of 3 years reported by RNs who worked in CCUs of 10 hospitals. The total number of self-reported PSIs that were analysed was 1017, as illustrated in Table 2.

**TABLE 2:** Types and frequencies of Patient Safety Incidents in selected critical care units of eThekwini district, KwaZulu-Natal.

Variable	*n*	%
**Death**		
Unnatural death (suicide, homicide, maternal, neonatal, procedure-related)	64	29
Death associated with a nosocomial infection (e.g. VAP)	58	26
**Procedure-related events**		
Surgery on wrong body part	11	5
Surgery on wrong patient	4	2
Wrong surgical procedure performed on patient	8	4
Unplanned return to operating room on admission	18	8
**Patient care-related events**		
Transfer from general care unit to a higher level, for example, high care or ICU	100	45
Length of stay for more than 10 days	110	49
Unplanned second presentation to department within 48 hours for the same condition	49	22
Return to emergency department or outpatients department for complication related to the last hospital admission	42	19
Disability associated with labour-related event	9	4
**Medication-related events**		
Allergic reaction	32	14
Drug interaction	43	19
**Blood product-related events**		
Blood transfusion reaction (fever, jaundice, urticaria, etc.)	41	18
Incorrect blood administered (blood to wrong patient)	10	5
**Hospital-related events**		
Multi-drug resistant organism (organism resistant to three or more antibiotics)	103	45
Intravenous site inflammation/catheter-related infections	106	47
Post-operative wound infection	55	47
Hospital-incurred patient incident, such as fall	35	16
Development of pressure sores	79	35
Patient abscondment	22	10
Infant discharged to wrong person, or missing infant	5	2
Patient with needle-stick injury	13	6
**Total**	**1017**	**-**

VAP, ventilator-associated pneumonias; ICU, Intensive care unit.

**FIGURE 2:**
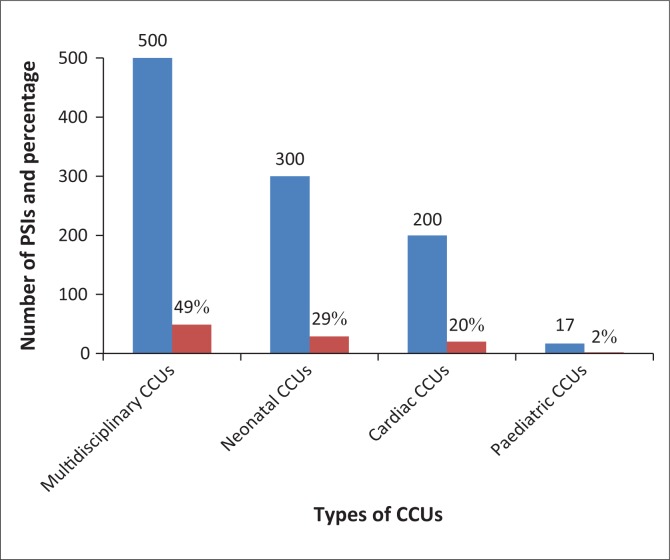
Frequencies of Patient Safety Incidents different types of selected critical care units of eThekwini district, KwaZulu-Natal (*n* = 1017).

High rates of PSIs, with increased length of stay, were observed in multidisciplinary CCUs (500 [49%]), neonatal CCUs (300 [29%]) and cardiac CCUs (200 [20%]), compared with less incidents in paediatric CCUs (17 [1.7%]), as illustrated in Figure 2.

It was also observed that ventilator-associated pneumonias (VAP) were the major cause of death in neonatal CCUs (30% [*n* = 67). Multi-drug resistance (80% [*n* = 814]) and development of bed sores (78% [*n* = 793]) were the most reported PSIs in multidisciplinary CCUs.

The chi-square tests were performed to assess the differences between the participating hospitals, both the government and private hospitals. There was no statistical significance (*p* > 0.05) on comparison between these hospitals, as similar frequencies of PSIs were observed, indicating that the results reflected a general distribution pattern in CCUs.

### Patient outcomes

The extent of PSIs, from insignificance to catastrophic, was classified into five categories in accordance with the Australian/New Zealand Standard AS/NZS ISO 31000:2009 (Hooper & Tibballs 2014). Each category is explained by the severity of patient outcome, as illustrated in Table 3.

**TABLE 3:** Classification of Patient Safety Incidents according to severities.

Variable	Incidents
Insignificant	No injuries; low financial loss
Minor	Treatment required, no increase in length of stay or readmission; minor financial loss
Moderate	Temporal injury, increased length of stay or readmission; medium financial loss
Major	Permanent injury, increased length of stay or readmission; major financial loss
Catastrophic	Death; huge financial loss or threat to goodwill

*Source:* Hooper, A. & Tibballs, J., 2014, ‘Comparison of a trigger tool and voluntary reporting to identify adverse events in a paediatric intensive care unit’, *Anaesthesia and Intensive Care* 42(2), 199. https://doi.org/10.1177/0310057X1404200206

**FIGURE 3:**
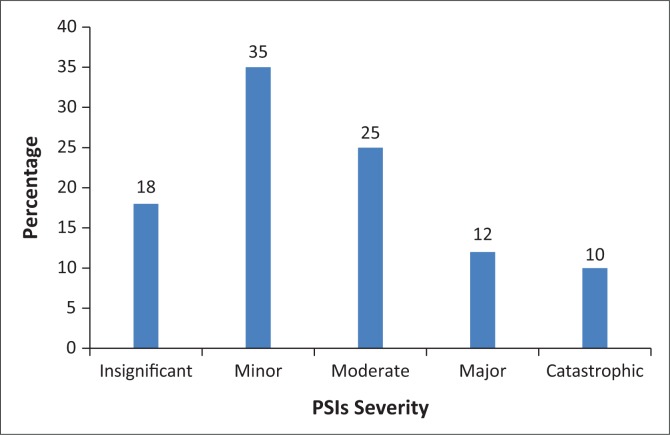
Severities of Patient Safety Incidents in participating hospitals.

The patient outcome revealed that of the 1017 reported PSIs, 18% (*n* = 70) were insignificant, 35% (*n* = 90) minor, 25% (*n* = 72) moderate, 12% (*n* = 32) major and 10% (*n* = 26) catastrophic, as illustrated in Figure 3. The majority of PSIs were minor and insignificant which required minor treatment, neither increase in the length of hospital stay nor readmission, and resulted in low or minor financial loss.

Blood-related incidents (5%) and medication-related events (7%) were more minor or insignificant, as most of the time the correction measures were successful. Procedure-related events (4%) were also minor or insignificant, as these PSIs hardly happened in CCUs, but if they occurred, they had a catastrophic outcome.

## Discussion

### Existence and utilisation of reporting system

The Institute of Medicine (IOM) released a report on patient safety, entitled ‘To Err Is Human’ (Ahmed et al. 2015; Ramya 2017; West & Eng 2014). In this report, the IOM defined safety as freedom from injury and error and further classified the types of errors into diagnostic, treatment, preventative and other categories (Heavner & Siner 2015; West & Eng 2014). Aligning itself with the WHO recommendations, South Africa formulated ‘National Guidelines for Patient Safety Incident Reporting and Learning System’ to guide health care system in dealing with patient safety incident reporting and to implement a nationwide, uniform PSI reporting system, whereby the health care practitioners could learn from the reported PSIs (NDoH 2017). This strategy was adopted at a provincial level in KZN DoH, which aligned itself with a broader vision encapsulated in NDoH (Mahomed, Moodley & Jinabhai n.d.).

In this study, the majority of respondents (84%) said they used the reporting system, but some indicated that they did not use the system. The reasons for not using the system included that the process was too long and complicated, non-reaction to the report, fear of litigation, being too busy and lack of feedback following incident reporting. Other reasons for non-compliance were fear of disciplinary action by the South African Nursing Council (SANC) and victimisation and ridicule by peers. A study conducted by Pepper and Slabbert (2011) asserts that South Africa is witnessing a sharp rise in medical malpractice litigations as patients have become more conscious of their rights. Various studies have confirmed that there are barriers that led to underreporting (Mjadu & Jarvis 2018; Moumtzoglou 2010). In one study, conducted in the United States with 13 participants, underreporting was because of lack of training on patient safety reporting and instructions, lack of reporter-friendly classifications and time constraints (Gong et al. 2015). According to Mjadu and Jarvis (2018), blaming the individual and threats of future litigation are predominant in health care. In addition, processes and systems of reporting barriers included lack of anonymity and/or confidentiality in reporting (Archer et al. 2017; Mjadu & Jarvis 2018).

It has also been observed in other studies that although both medical doctors and nurses were aware of the PSI reporting system, nurses reported more frequently than doctors (Moumtzoglou 2010; Schultz et al. 2014). The reasons for underreporting by doctors were fearfulness and mistrust of the reporting system, differences in what constitutes PSIs, poor training and attitude towards reporting. Schultz et al. (2014) recommend that to address some of the known barriers in using the system, repercussions of non-compliance should be non-punitive, protected, confidential and independent from regulations, provide timely feedback and be systems-oriented and voluntary.

The results of this study confirm the findings of previous studies which identified underreporting of PSIs as still a major problem (Gong et al. 2015; Hooper & Tibballs 2014). This study claims the total of 1017 of the reported PSIs during the 3-year period; however, some of these PSIs may have been missed because of underreporting.

### Types and frequencies of Patient Safety Incidents

There is a general consensus that PSIs during hospital care are a serious problem (Bashir et al. 2019; Guillod 2013; Hauck & Zhao 2011). According to Bauman and Hyzy (2014), the use of information and health technology has tremendous potential for improving the efficiency, cost-effectiveness, quality and safety of medical care. This study’s results confirmed findings similar to that of other studies reporting medical PSIs; adverse drug reactions, hospital-acquired infections, multi-drug resistance and development of pressure sores were the most reported PSIs in medical, general and multidisciplinary CCUs (Naessens et al. 2012). This evidence could be because of the nature of patients’ disease profile and hospital-acquired infections that are difficult to treat, especially because of lack of antibiotic stewardship and increased number of patients with HIV and AIDS.

Patient care-related incidents were associated with increased length of stay (more than 10 days) as a result of intravenous site inflammation/catheter-related infections, invasive procedures by nature, severity of the disease in progress, trauma and polypharmacy that could have exposed patients to PSIs. This study also confirms the findings, similar to that of other studies, that increasing the length of stay in neonatal units was the major cause of mortality and morbidity, not because of the underlying disease but because these patients were more exposed to PSIs’ occurrence (Ahmed et al. 2015; Hauck & Zhao 2011). These results also suggest that there could be underlying conditions, as confirmed by Naessens et al. (2012), that patients with comorbid conditions and a higher admissions severity were likely to suffer from PSIs during their hospital stay.

There was also variation in the types and categories of PSIs reported by different hospitals depending on the policy of particular hospitals regarding reporting of PSIs. Furthermore, the results revealed that better reporting of PSIs was not necessarily because of unsafe environments, but it was because some hospitals that were using the web-based system found it more effective as staff did not have to divulge their identity.

### The patient outcome, indicating the severity of Patient Safety Incidents

The majority of PSIs were minor and insignificant, and hence required minor treatment, neither increased the length of stay in hospital nor readmission, thus minimising financial losses. Blood-related and medication-related events formed part of this category as most of the time the corrective measures were successful. However, this study revealed that the occurrence of PSIs was a serious problem as these are associated with increase in morbidity and mortality rates. Of the 1017 PSIs, 47% were classified as moderate, major and catastrophic, which indicated that occurrence of PSIs was still high in CCUs and was associated with increased length of stay, as reported in other studies (Ahmed et al. 2015; Roque et al. 2016; Sommella et al. 2014).

## Conclusion

This study reveals that the occurrence of PSIs in CCUs is still high and is of a serious nature, which affects quality patient care and patient safety. It was also noted that the reporting system for PSIs was not effectively utilised, mainly because of fear of litigation and disciplinary action. Implementation of uniform national reporting system of PSIs is crucial to improve quality patient care in CCUs.

### Limitations

The findings of this study cannot be generalised to other settings as only one district of KZN was studied.

### Recommendations

Although there are guidelines that have been stipulated by the NDoH and KZN DoH, there is no evidence in the reduction of PSIs in CCUs. Development of a simplified efficient model that ensures standardisation of reporting of PSIs in CCUs is recommended. Such a model should be non-punitive, non-confrontational and supported by adequate training of health care practitioners. This study was conducted in one district of one province; a study in more districts of all provinces of South Africa could provide a national perspective on reporting of PSIs.
