# Erratum: Analysis of Patient Safety Incident reporting system as an indicator of quality nursing in critical care units in KwaZulu-Natal, South Africa

**DOI:** 10.4102/hsag.v26i0.1559

**Published:** 2021-07-29

**Authors:** Thusile M. Gqaleni, Busisiwe R. Bhengu

**Affiliations:** 1 School of Nursing and Public Health, University of KwaZulu-Natal, Durban, South Africa

In the version of this article initially published, Gqaleni, T.M. & Bhengu, B.R., 2020, ‘Analysis of Patient Safety Incident reporting system as an indicator of quality nursing in critical care units in KwaZulu-Natal, South Africa’, *Health SA Gesondheid* 25(0), a1263. https://doi.org/10.4102/hsag.v25i0.1263, the article section was given incorrectly. The correct section should be Original Research instead of Review Article.

This correction does not alter the study’s findings of significance or overall interpretation of the study’s results. The publisher apologises for any inconvenience caused.

